# Intraductal Injection of Adenoviruses to Perform Lineage Tracing in the Mammary Gland

**DOI:** 10.1007/s10911-025-09584-6

**Published:** 2025-07-07

**Authors:** Xueqing Chen, Sen Han, Dongyi Zhao, Zhe Li

**Affiliations:** 1 Division of Genetics, Department of Medicine, Brigham and Women’s Hospital, Boston, MA 02115 USA; 2 Department of Medicine, Harvard Medical School, Boston, MA 02115 USA

**Keywords:** Pulse-chase Lineage Tracing, Adenovirus, Breast cancer, Cellular Origin, Cell-of-origin, Mammary Gland, Mammary Epithelial Cell, Intraductal Injection

## Abstract

Lineage tracing is a fundamental tool in developmental biology and cancer research, providing critical insights into cell fate decisions, tissue homeostasis and tumor initiation. The mammary gland is a highly dynamic organ with a complex cellular hierarchy, making it an ideal system for lineage-tracing studies. Classic approaches, such as tamoxifen-inducible CreER/loxP recombination, have significantly advanced our understanding of mammary epithelial cell (MEC) differentiation, homeostasis, and transformation. However, these methods have limitations, including potential effects of tamoxifen on estrogen signaling, low mammary gland specificity, and the requirement for transgenic model creation and mouse breeding. Adenovirus-Cre (Ad-Cre)-based lineage tracing has emerged as a powerful alternative, enabling rapid and organ-specific recombination. This review provides a comprehensive evaluation of the Ad-Cre approach in mammary gland biology, comparing its efficiency, specificity, and technical advantages over the CreER-based method. We discuss applications of Ad-Cre intraductal injection-based lineage tracing in mapping MEC fates, identifying the cellular origins of breast cancer, and modeling tumor progression. Additionally, we highlight its ability to induce genetic marking at a clonal level, facilitating precise investigations into MEC plasticity and tumor cell heterogeneity. Despite its advantages, Ad-Cre lineage tracing also presents challenges, such as low cell-targeting efficiency and potential effect on the mammary gland immune microenvironment. Future advancements, including the integration of CRISPR-based barcoding, may further enhance its utility for high-resolution fate mapping. By summarizing recent advancements and comparative analyses, this review underscores the significance of Ad-Cre lineage tracing as a versatile and powerful tool in mammary gland biology and breast cancer research.

## Introduction

Pulse-chase lineage tracing is a fundamental technique in molecular and cellular biology employed to track the fate of specific cell types over time. This method has been extensively used to study cell proliferation, differentiation, and migration within living organisms. It is particularly valuable in developmental biology, cancer research, and stem cell studies, as it provides insights into how targeted cells contribute to tissue formation, homeostasis, and the process of tumor initiation, progression and metastasis. The core principle of pulse-chase lineage tracing involves labeling a specific population of cells at a given time and subsequently monitoring these cells and their descendants over time to track their cell fates during cell division, migration and differentiation.

The core process of pulse-chase lineage tracing begins with labeling a specific group of cells with a distinguishable marker, such as fluorescent dyes or genetically inducible reporters. Early applications of this approach were largely limited to direct observations, due to challenges in the labeling efficiency. These include monitoring cell divisions in certain transparent vertebrate embryos or invertebrates, such as zebrafish embryos [1] or *C.elegans* [2]. While providing real-time insights, its application was limited to organisms that were amenable to such observations [3]. Early methods of lineage tracing also utilized fluorescent reporters or dyes, which were introduced into cells via microinjection or electroporation [4]. However, these techniques presented drawbacks, such as dye dilution over successive cell divisions and the potential for contamination to neighboring cells, limiting their long-term utility.

Recent advancements in lineage tracing include the use of photoactivatable fluorescent proteins to label embryonic cells [5] and the broader adaptation of genetically inducible strategies [6]. Modern pulse-chase lineage tracing techniques often employ genetically engineered systems such as Cre/loxP recombination [7, 8] and Tet-On/Tet-Off systems [9], which provide more precise temporal and spatial control over cell labeling [10]. In the Cre/loxP system, a transcriptional “Stopper” cassette, typically inserted into the mouse *Rosa26* locus as part of a Cre reporter, is flanked by loxP sites. A Cre recombinase enzyme, usually fused to the estrogen receptor (CreER, or CreERT2, which is the second generation of CreER fusion commonly used in mammary gland biology), is expressed from a cell type-specific promoter [11]. Upon activation by tamoxifen, CreER recombines the loxP sites, leading to excision of the floxed “Stopper” cassette and permanent genetic labeling (i.e., in the form of expression of the reporter gene downstream of the floxed “Stopper”) of the target cells and their progeny by the now activated Cre reporter [12]. Similarly, the Tet-On/Tet-Off system uses tetracycline or doxycycline to control the expression of either reporters [13] or Cre recombinase, which subsequently activates a Cre reporter [14]. Both genetically inducible tools are widely used in a variety of research in developmental biology, oncology and stem cell research to study cell plasticity, migration and reprogramming in tissue homeostasis and regeneration [15].

The mammary epithelium consists of luminal and basal mammary epithelial cells (MECs), which are organized in a hierarchy. Based on the classic cleared fat pad transplantation assay, it was initially demonstrated that a single mammary stem cell (MaSC), which is a basal cell, can generate all types of MECs upon transplantation [16, 17]. During development, MaSCs are also present in the fetal mammary gland, where fetal MaSCs (fMaSCs) exhibit multipotency and can reconstitute the entire mammary epithelium upon transplantation [18]. To complement the transplantation approach, pulse-chase lineage tracing has been used to investigate the MEC hierarchy and its stem/progenitor cells [19]. Lineage tracing revealed that fMaSCs are multipotent, giving rise to both luminal and basal MEC progenies in adults [20, 21]. However, postnatally, the luminal and basal lineages appear to be primarily maintained by their respective lineage-restricted stem/progenitor cells [12, 22–24]. While in situ lineage tracing shows that most postnatal basal cells are lineage-restricted, they can become multipotent after transplantation. Under physiological conditions, the transition from multipotency to unipotency can occur as early as during embryonic development [21, 25]. Furthermore, the luminal lineage comprises estrogen receptor (ER)^+^ luminal cells, which are typically ductal cells, as well as ER^−^ luminal cells, which are largely alveolar luminal cells and their progenitors. Lineage tracing studies have demonstrated that these two luminal sub-lineages may also be maintained by their corresponding sub-lineage-restricted progenitors [26–29].

Lineage tracing studies of MECs so far have primarily relied on Cre/loxP genetic approaches, using either tamoxifen-induced CreERT2 activation or doxycycline-induced Cre expression for pulse labeling of target MECs. To complement these widely used conditional strategies, our group developed an alternative Cre-based lineage tracing method for MECs involving intraductal injection of Cre-expressing adenoviruses (Ad-Cre) under the control of various MEC subset-specific promoters. In this review, we outline the principles of this lineage tracing approach and its applications in studying the in vivo fate of MEC subpopulations and in inducing mammary tumor initiation. Additionally, we compare this novel approach with existing lineage tracing techniques.

## In vivo Kinetics of Adenovirus-encoded Protein

The core principle of pulse-chase lineage tracing is to label a target cell population only within a defined time frame (i.e., pulse). In the Cre/loxP system, this is typically achieved by transiently inducing Cre recombinase activity, such as through tamoxifen-induced CreER activation or doxycycline-induced Cre expression. Alternatively, transient Cre expression can also be achieved via an adenovirus. Adenoviruses are double-stranded DNA viruses that persist episomally in host cells without integrating into their genomes. Previous research has shown that during mitosis, adenoviral genomes can attach to condensed chromatin, ensuring their equal distribution to daughter cells after division [30]. However, since the Ad-Cre viruses used for lineage tracing are replication-deficient, they become diluted over time in infected cells and their progeny, thereby restricting Cre expression to a relatively short period.

To assess the in vivo kinetics of adenovirus-encoded proteins in the mammary gland, green fluorescent protein (GFP) protein expression from a *CMV* promoter-driven adenovirus was used as a surrogate. Following a single intraductal injection of *CMV-GFP* adenovirus into adult female mice (~ 2 months of age), it was found that GFP^+^ cell levels in the injected mammary gland peaked at day 2 post-injection, declined rapidly within days, and reached nearly zero by two weeks [12]. This transient transgene expression pattern has also been observed in other studies (e.g [31]).,. If the half-life of Cre protein from the injected Ad-Cre is comparable to that of GFP, it is reasonable to assume that the Cre recombinase activity in the injected mammary gland may persist for approximately two weeks post-injection. Notably, the *CMV* promoter used in the aforementioned study (to drive GFP expression) is significantly stronger than those driving Cre expression in specific MEC subpopulations (e.g., *Krt8*, *Krt5*, see below). Consequently, Cre protein levels from these MEC subset-specific promoters may be lower, potentially leading to an even shorter window of effective genetic marking. In contrast, while adeno-associated viruses (AAVs) also do not integrate into the host cell genome, they enable much longer-lasting transgene expression compared to adenoviruses [31], making them suitable for gene therapy but not ideal for pulse-chase lineage tracing. Similarly, lentiviruses integrate into the host genome and facilitate stable, long-term transgene expression, which also limits their applicability for pulse-chase lineage tracing.

## Intraductal Injection of Ad-Cre to Target Different MEC Subsets

The commonly used Ad-Cre virus expresses Cre under the control of the *CMV* promoter, enabling Cre-mediated recombination across all MEC subsets [12] (*Ad-CMV-Cre*, Table 1). The adenoviral vector used here can accommodate DNA inserts up to 8 kb [30]; thus, replacing the *CMV* promoter with a cell type-specific promoter (e.g., under 7 kb) could, in theory, restrict Cre expression and Cre-mediated recombination to a particular cell type. To test this possibility, several newly generated and existing Ad-Cre viruses, each driven by different MEC subset-specific promoters, were evaluated via intraductal injection (Table 1).

**Table 1:** Promoter-specific Ad-Cre viruses used in intraductal injection

Type of Ad-Cre	Promoter	MEC subset specificity	Level of Cre expression*	Reference
*Ad-CMV-Cre*	Viral *CMV*	Luminal, basal	High	[12]
*Ad-K8-Cre*	Mouse *Krt8*	Luminal	Medium	[12]
*Ad-K14-Cre*	Human *KRT14*	Basal, some luminal	Medium	[12]
*Ad-K5-Cre*	Bovine *Krt5* [32]	Basal	Medium	[32]
*Ad-Wap-Cre*	Mouse *Wap*	Alveolar luminal and luminal progenitor	Low	[27]
*Ad-Esr1-Cre*	Mouse *Esr1* [28]	ER^+^ luminal?	Very low?	

*Level of Cre expression is estimated based on the percentage of YFP^+^ cells detected by FACS analysis in the mammary glands of *R26Y* reporter mice several days after intraductal injection of the corresponding Ad-Cre viruses (High: >5%, Medium: ~0.1%-2%, Low: ~0.01%-0.1%, Very low: <0.01%)

To target luminal MECs, an Ad-Cre virus was engineered to express Cre under the control of the promoter of a pan-luminal keratin marker gene, *Krt8* (*K8*) (Table 1). Intraductal injection of the resulting *Ad-K8-Cre* virus into female mice carrying the conditional *Rosa26-loxP-Stopper-loxP-YFP* (*R26Y*) reporter [33] resulted in yellow fluorescent protein (YFP) reporter expression specifically in luminal MECs, as confirmed by both fluorescence-activated cell sorting (FACS) analysis and immunostaining [12].

For basal MECs, an initial attempt using an Ad-Cre virus driven by the promoter of a basal keratin marker gene, *KRT14* (*K14*), led to YFP expression in both basal MECs and a subset of luminal MECs [12] (Table 1). A subsequent effort tested *Ad-K5-Cre*, previously used in lung cancer studies [34], which employs a different basal keratin gene promoter, *Krt5* (*K5*) (Table 1). This virus achieved more precise targeting of the basal MEC lineage for Cre-mediated recombination [32].

Since the luminal lineage comprises both ER^+^ and ER^−^ luminal cells, and *Ad-K8-Cre* targets both subsets, developing cell type-specific Ad-Cre viruses for each subset remains of great interest. ER^−^ luminal cells largely consist of alveolar luminal cells and their progenitors [i.e., often referred to as luminal progenitors (LPs)]. Such LPs are of particular interest as they may serve as cells-of-origin of multiple breast cancer subtypes, including basal-like breast cancer [35–37]. To specifically target al.veolar luminal cells and LPs, a novel Ad-Cre virus, *Ad-Wap-Cre*, was developed using the whey acidic protein (*Wap*) promoter [27] (Table 1). In nulliparous female mice, the *Wap* promoter is transiently active during estrus [38]; thus, intraductal injection of *Ad-Wap-Cre* into virgin female mice successfully targeted the (alveolar) LP subpopulation [27].

To target ER^+^ luminal MECs, a recent study employed the *Esr1* promoter to drive rtTA expression, coupled with doxycycline induction, demonstrating that ER^+^ luminal cells are also self-sustained by their sub-lineage-restricted stem/progenitor cells [28]. Using the same *Esr1* promoter, we generated a new Ad-Cre virus, *Ad-Esr1-Cre* (Table 1). However, preliminary tests of its lineage specificity have been inconclusive due to extremely low targeting efficiency in vivo, though it is unclear whether this is due to low Cre expression or a low titer of the adenovirus. Previously, we showed that intraductal injection of *Ad-K8-Cre* into *R26Y* female mice carrying conditional knockout alleles of *Trp53* (i.e., *Trp53*^*L/L*^;*R26Y*) initially led to expansion of p53-deficient ER^+^ luminal cells [39]. Intraductal injection of *Ad-Esr1-Cre* into the same *Trp53*^*L/L*^;*R26Y* mice resulted in a small but detectable ER^+^ luminal population (by FACS), suggesting that *Ad-Esr1-Cre* may have the correct lineage specificity. However, its low targeting efficiency likely limited detection, with p53-loss driving the expansion of ER^+^ luminal cells to a detectable level (HS, XC, and ZL, unpublished observation).

## Intraductal Injection of Ad-Cre to Map in vivo Fates of Different MEC Subsets

A recent wave of Cre/loxP-based lineage tracing studies have provided novel insights into the development, hierarchy, and maintenance of MECs in vivo (e.g., [14, 20–26, 28, 29, 40, 41]). These studies primarily relied on transiently induced Cre-mediated recombination, such as tamoxifen-induced CreERT2 activation or doxycline-induced Cre expression, for pulse-chase lineage tracing. Since intraductal injection of various Ad-Cre viruses also leads to transient Cre expression, they could, in theory, be used similarly to map the in vivo fates of MECs.

Indeed, lineage tracing using intraductal injection of *Ad-K8-Cre* or *Ad-K14-Cre* into *R26Y* female mice demonstrated that luminal and basal lineages are largely self-sustained, consistent with findings from tamoxifen/CreERT2-based lineage tracing studies [12]. While previous studies suggest the presence of long-lived LPs that maintain the luminal lineage, their precise identity and role in breast cancer development remain unclear [42]. To specifically target luminal MECs in the alveolar lineage, *Ad-Wap-Cre* was delivered via intraductal injection into nulliparous female mice [27]. This pulse-chase approach allowed researchers to track the fate of MECs marked by *Ad-Wap-Cre*, particularly during pregnancy, lactation, and involution. The results revealed that in nulliparous female mice, the marked MECs were enriched with CD61^+^ alveolar progenitors. During pregnancy and lactation, these progenitors differentiated into CD61^−^ alveolar luminal cells, responsible for milk production. Notably, the *Ad-Wap-Cre*-labeled cells remained long-term and even after ovariectomy, indicating their ability to self-maintain independently of ovarian hormones [27].

It is important to note that lineage tracing studies using these lineage-restricted genetic tools with cell type-specific promoters may not fully capture the in vivo fates of different MEC subsets. In fact, neutral lineage tracing studies based on the use of the ubiquitous *Rosa26-CreERT2* mouse line coupled with the *Rosa26-Confetti* reporter have shown that mammary stem and progenitor cells exhibit extensive redundancy and heterogeneity, with these diverse populations working together to sustain the mammary epithelium long-term [43, 44]. In principle, neutral lineage tracing can also be performed in *Rosa26-Confetti* reporter mice upon intraductal injection of *Ad-CMV-Cre*. By using a lower titer of *Ad-CMV-Cre*, MECs can be genetically marked at a clonal density for unbiased lineage tracing. However, there are several considerations: First, while intraductally injected fluid reaches all regions of the mammary ductal tree [12, 32], it is unclear whether the similarly injected *Ad-CMV-Cre* can effectively and unbiasedly mark MECs at different regions. Second, although both luminal and basal MECs can be targeted by *Ad-CMV-Cre* [12], the injected adenovirus is likely to preferentially infect luminal cells, given its location within the lumen, potentially skewing the labeling toward the luminal population. Lastly, due to technical limitations, successful intraductal injections can only be performed in female mice aged 3–4 weeks or older, preventing lineage tracing of MECs in younger mice (e.g., during fetal development).

## Intraductal Injection of Ad-Cre to Study Mammary Tumor Initiation, Progression, and Metastasis

**Fig. 1:**
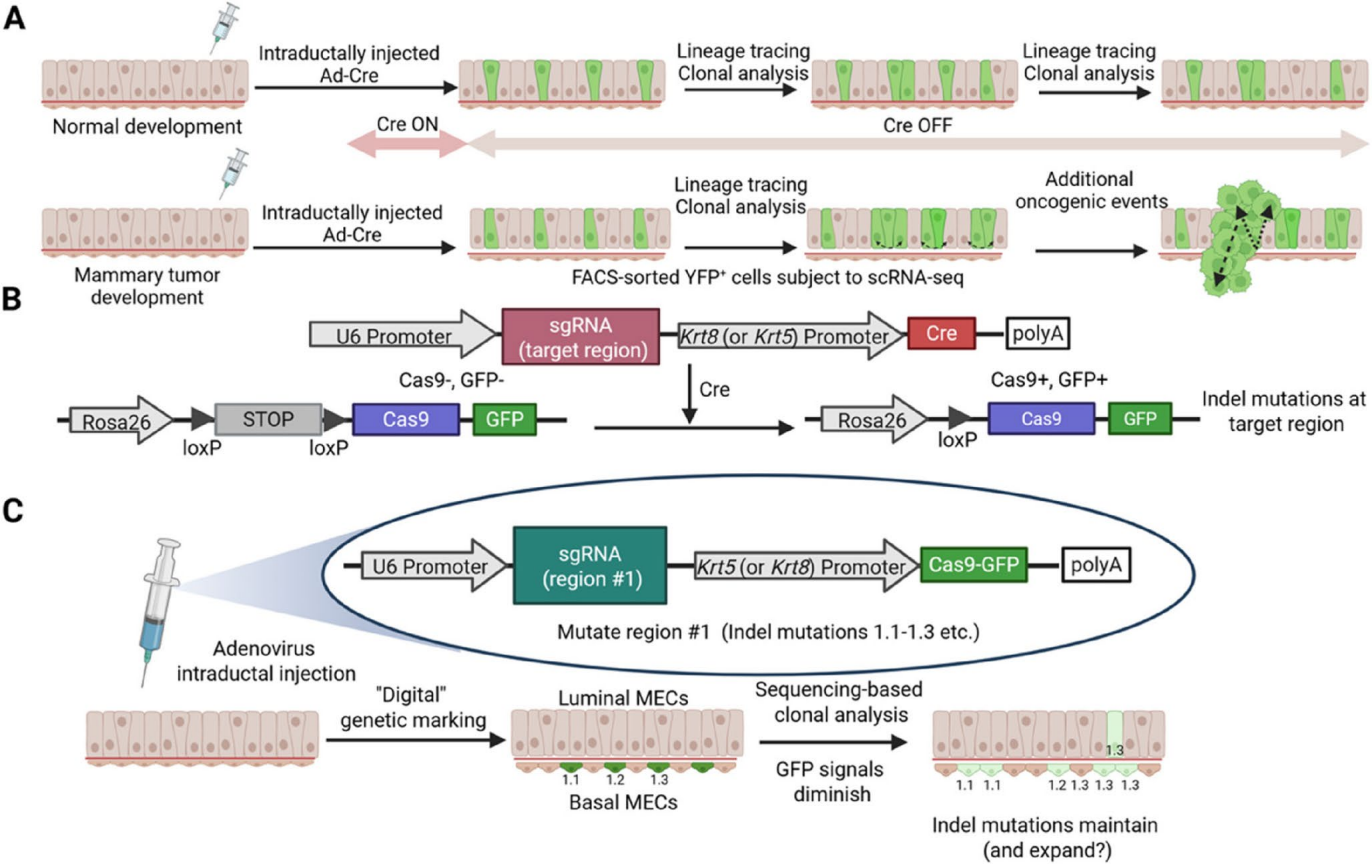
Schematic representation of adenovirus intraductal injection-based lineage tracing (created with BioRender.com). **A** Pulse-chase lineage tracing following intraductal injection of Ad-Cre. Green cells represent YFP-marked MECs and their progression during normal homeostasis or mammary tumor development.** B** Schematic diagram illustrating the adenoviral vector and reporter mouse line used for Cre/loxP-based “analog” (e.g., GFP) lineage tracing and CRISPR/Cas9-based “digital” barcoding. **C** Diagram showing an adenoviral vector encoding both Cas9 and single guide RNA (sgRNA) within the same virus. CRISPR-induced mutations (e.g., 1.1, 1.2, 1.3, etc.) serve as barcodes at a designated genomic locus (region #1). While adenovirus-encoded GFP signals (and Cas9 expression) fade over time, the newly introduced genomic mutations in the initially GFP^+^ MECs remain stable and are inherited by their daughter cells

Various Ad-Cre viruses targeting different MEC subsets are valuable not only for mapping MEC lineage fates in vivo but also for investigating their potential roles as cells-of-origin in breast cancer (Fig. 1A). Previous research using *Ad-K8-Cre* in mice carrying the *Etv6-NTRK3* fusion gene, which is associated with human secretory breast carcinoma [45], demonstrated that Cre-mediated recombination in luminal cells led to the formation of heterogeneous mammary tumors [12]. This finding suggests that luminal MECs can serve as cells-of-origin for diverse tumor subtypes.

Since *Ad-K8-Cre* targets both ER^+^ and ER^−^ luminal MECs, a more specific approach was needed to study ER^−^ luminal cells as potential cells-of-origin. To address this, the alveolar luminal-specific *Ad-Wap-Cre* was used to induce mammary tumor initiation in mice carrying the *Etv6-NTRK3* fusion gene [27]. Notably, tumor characterization in these models revealed three distinct tumor types in *Ad-K8-Cre*-injected mice (i.e., Type 1: KRT8^+^KRT14^−^ luminal tumor cells surrounded by KRT14^+^KRT8^−^ basal tumor cells; Type 2: KRT8/KRT14 double-positive; Type 3: KRT8^+^KRT14^−^ luminal only). In contrast, *Ad-Wap-Cre*-injected mice only developed the first two tumor types (i.e., Type 1 and 2) [12, 27]. These findings suggest that, under the influence of the same Etv6-NTRK3 oncoprotein, mammary tumors with basal transdifferentiation may originate from alveolar LPs, whereas mammary tumors that are strictly luminal may arise from ER^+^ luminal cells (or other ER^−^ ductal luminal cells).

Previous studies have shown that ectopic expression of the Pik3ca^H1047R^ mutant in either luminal or basal MECs can induce multipotency in both lineages [46, 47]. Consistent with this, intraductal injection of *Ad-K5-Cre* (targeting basal MECs) or *Ad-K8-Cre* (targeting luminal MECs) to Pik3ca^H1047R^ conditional knockin mice led to mammary tumor development in both cases [48]. Collectively, these Ad-Cre-based studies support the idea that dominant oncoproteins, such as Etv6-NTRK3 or Pik3ca^H1047R^, can transform multiple MEC subsets to initiate mammary tumorigenesis.

Intraductal injection of Ad-Cre viruses can also induce the loss of tumor suppressor genes, leading to mammary tumor development. One study demonstrated that intraductal injection of *Ad-K8-Cre* to *Trp53*^*L/L*^;*R26Y* female mice resulted in mammary tumors with 100% penetrance, with most tumors resembling human Claudin-low breast cancers [39]. Interestingly, a similar outcome was observed when *Ad-Wap-Cre* was injected into *Trp53*^*L/L*^;*R26Y* female mice (XC and ZL, unpublished observation), raising a possibility that Claudin-low mammary tumors in these models may originate from alveolar LPs.

In another study [49], intraductal injection of *Ad-K8-Cre* to *Trp53*^*L/L*^;*Brca1*^*L/L*^;*R26Y* female mice also led to mammary tumor development with full penetrance. However, in contrast to the Claudin-low tumors observed with *Trp53*-loss alone, the majority of tumors in this model (i.e., with additional loss of *Brca1*) were basal-like. This observation was further confirmed in a recent study, which demonstrated that inducing the loss of *Brca1* and *Trp53* in mammary ductal epithelium via *Ad-CMV-Cre* injection resulted in the development of basal-like, hormone receptor (HR)-negative mammary tumors [50]. Interestingly, induced loss of *Rb*, *Trp53* and *Brca1*, or the combined loss of *Rb* and *Trp53*, led to the development of HR^+^ (e.g., positive for ER) luminal ductal carcinoma [50]. This suggests that the HR status of the resulting tumors was primarily determined by the loss of *Rb*, rather than *Brca1* status [50]. Collectively, these findings indicate that the loss of specific tumor suppressors can dominantly shape mammary tumor subtypes, potentially through interactions with distinct MEC subpopulations.

Since intraductally injected Ad-Cre viruses target MECs at a clonal level, the introduction of conditional Cre-reporters such as *R26Y* enables tracing of their malignant progression and mapping of their aberrant cell fates in vivo (Fig. 1A). Upon intraductal injection of *Ad-K8-Cre* to *Trp53*^*L/L*^;*R26Y* or *Trp53*^*L/L*^;*Brca1*^*L/L*^;*R26Y* female mice, and by following the YFP-marked cells, it was observed that p53-deficient and p53/BRCA1-deficient premalignant MECs exhibited different kinetics of clonal expansion [39, 49]. Importantly, by sorting YFP-marked p53/BRCA1-deficient premalignant MECs and performing single cell RNA-sequencing (scRNA-seq) (Fig. 1A), researchers found that these mutant MECs largely consisted of aberrantly expanding alveolar LP-like cells that underwent dynamic luminal to basal/mesenchymal cell fate changes [49].

Similarly, a recent study demonstrated that intraductal injection of *Ad-K8-Cre* to mammary ducts of *Lats1/2*-floxed female mice resulted in the development of mammary tumors displaying luminal-basal plasticity and features of epithelial-mesenchymal transition [51]. LATS1 and LATS2 (LATS1/2) are Hippo pathway kinases that inhibit YAP/TAZ nuclear translocation and transcriptional activity and function as potential tumor suppressors in breast cancer [52]. Fate mapping revealed that loss of LATS1/2 in luminal MECs led to emergence of aberrant KRT8^+^KRT14^+^ premalignant cells [51].

Beyond mapping aberrant luminal-to-basal/mesenchymal cell fate changes, intraductal induction of *Ad-K5-Cre* into the above-described Pik3ca^H1047R^ conditional model also uncovered aberrant basal-to-luminal cell fate transitions [48]. Notably, by incorporating conditional knockout alleles for *Kdm6a* and performing lineage tracing coupled with scRNA-seq, this study further demonstrated that the loss of the COMPASS-like complex, of which the *Kdm6a*-encoded histone demethylase, KDM6A, is a key component, accelerated the conversion of basal MECs to aberrant alveolar-like luminal cells [48].

In addition to modeling mammary tumor initiation by directly activating oncogenic events in specific MEC subpopulations, intraductal injection of Ad-Cre can also be utilized to trace the cellular origin of mammary tumors in transgenic mouse models and to investigate how the induced loss of specific genes influences mammary tumor progression and metastasis. The *MMTV-PyMT* transgenic mouse model develops mammary tumors resembling human luminal B breast cancer [53]. In one study, pulse-chase lineage tracing was performed using intraductal injection of *Ad-Wap-Cre* into *MMTV-PyMT; R26Y* female mice. By tracking YFP-marked cells, researchers found that each *PyMT* tumor originated from a single cell-of-origin, either marked by *Ad-Wap-Cre* (YFP^+^) or not, indicating that *Ad-Wap-Cre*-marked alveolar LPs are one of the cellular origins of luminal mammary tumors in this model [27].

In another study, intraductal injection of *Ad-K8-Cre* was performed on *MMTV-PyMT; R26Y* female mice carrying conditional knockout alleles of *Lsd1* (i.e., *MMTV-PyMT; Lsd1*^*L/L*^;*R26Y*). Since *PyMT* tumors are K8^+^ luminal tumors, Cre expression in *Ad-K8-Cre*-infected tumor cells disrupted *Lsd1*. By tracking YFP-marked primary and metastatic tumor cells, it was found that while LSD1-loss did not significantly alter primary tumor development, it led to increased lung metastasis, suggesting that LSD1 may play a role in suppressing tumor metastasis [54].

## Comparison with Other Cre/loxP-based Lineage Tracing Approaches

Compared to other existing Cre/loxP-based lineage tracing methods, the Ad-Cre intraductal injection approach appears to most closely resemble the tamoxifen/CreERT2 approach in both targeting efficiency and available tools. Additionally, in the tamoxifen/CreERT2 system, significant recombination of reporter alleles has been observed even weeks after a pulse tamoxifen treatment, depending on the dose administered [55]. Similarly, a single intraductal injection of Ad-Cre viruses may result in Cre protein expression lasting for two weeks [12], suggesting that the kinetics of Cre-mediated recombination is comparable to that of the tamoxifen/CreERT2 system. Supporting this notion, breast cancer mouse modeling studies using both approaches have reported nearly identical tumor latencies and phenotypes, with the two methods often being used interchangeably [39, 48, 51].

However, the Ad-Cre intraductal injection approach also offers several advantages (Table 2). First, it avoids the use of tamoxifen. Since tamoxifen is a selective estrogen receptor modulator (SERM), its activation of CreERT2 activity can potentially influence mammary tumor development in mice by altering estrogen signaling in the mammary gland [26, 56]. Additionally, tamoxifen may have unintended effects on the cells of interest, such as inducing apoptosis [57]. Second, CreERT2 or Cre transgenic approaches may lead to cancer induction in other tissues if the promoter driving CreERT2 or Cre expression is active elsewhere. In contrast, intraductal injection of Ad-Cre restricts Cre expression to MECs, thus avoiding this potential problem. Third, generating a new promoter-specific Ad-Cre virus is easier than creating a new transgenic mouse line, giving researchers more flexibility to quickly perform lineage-tracing studies for different MEC subsets. Related to this, the Ad-Cre intraductal injection approach eliminates the need for generating new mouse models, thereby reducing breeding time and costs. Fourth, because Ad-Cre only permits transient Cre expression in vivo, it is well-suited for pulse-chase lineage tracing and minimizes concerns about non-specific mutations caused by constitutively expressed Cre [58]. Lastly, when modeling breast cancer in mice, Ad-Cre can be titrated to target only a small number of MECs in vivo. This allows mutant MECs targeted by Ad-Cre to evolve through interactions with their wild-type neighbor MECs (e.g., competing), as well as stromal cells and immune cells, thus providing a more accurate model of cancer initiation in humans.

**Table 2:** Comparison between Ad-Cre intraductal injection and tamoxifen/CreERT2 activation approaches

Approach	Ad-Cre intraductal injection	CreERT2 activation by intraperitoneal (IP) injection of tamoxifen
Effect on estrogen signaling	No effect	Tamoxifen affects estrogen signaling
Organ specificity	High (restricted to the injected mammary gland)	Low (CreERT2 activity in other organ if its promoter is active there)
Flexibility	Easier to design new promoter-specific Ad-Cre viruses; no need for new transgenic mouse lines	May require generation of new CreERT2 transgenic mouse lines for different targets
Time and cost	Reduces need for mouse breeding; faster and more cost-effective	Requires more breeding and maintenance of transgenic lines
Duration of Cre/CreERT2 activity	Transient Cre expression, may last for up to two weeks	High concentrations of IP injected tamoxifen may activate CreERT2 for weeks
Cell-targeting efficiency	Low	Low to medium
Effect on immune cells	May alter the “baseline” immune landscape of the injected mammary gland long-term	Less effect

## Injected Ad-Cre Induces Immune Response Transiently and Alters Immune Cell Landscape long-term

A key concern with any virus-based gene-editing approach in vivo is the potential to elicit immune responses, both innate and adaptive, due to the inherent immunogenicity of viral particles [59–61]. While AAVs and lentiviruses generally exhibit low immunogenicity and induce only mild immune reactions, adenoviruses are more immunogenic and can provoke a stronger immune response compared to AAVs and lentiviruses [60, 61]. Histology analyses, including our own and those of others, have previously reported no significant inflammatory response in the injected mammary glands at later time points post-injection [12, 62]. However, although the immune responses triggered by injected adenoviruses are transient, it remains possible that immunological footprints from the viral infection could persist within the target tissues, such as the mammary gland, for an extended period.

To test this, we further characterized immune cells in the mammary glands of the *Trp53*^*L/L*^;*R26Y* mouse model at various time points after intraductal injection of *Ad-K8-Cre* by FACS analysis and compared immune cell changes with those in the *K8-CreER; Trp53*^*L/L*^;*R26Y* mouse model following tamoxifen induction (SH and ZL, unpublished observation). In the adenovirus-injection model, we observed a strong, presumably antiviral immune response shortly after viral infection. Despite the transient nature of this initial immune reaction, the infected mammary glands exhibited higher levels of leukocyte infiltration than those in the tamoxifen-induced model at later time points. This difference was primarily driven by CD8^+^ T cells, which displayed an activation phenotype and persisted at high levels long-term, expressing tissue-resident T cell markers (e.g., CD103). In contrast, the tamoxifen-induced model showed lower baseline CD8^+^ T cell infiltration, with their upregulation and activation occurring only upon p53-loss induction. Notably, in the *Ad-K8-Cre*-induced model, the adenovirus-driven immune response may have masked a similar p53-loss-induced CD8^+^ T cell change due to the already elevated baseline CD8^+^ T cell infiltration. Additionally, the influx of CD8^+^ T cells may have “compressed” the composition of other immune cell populations (e.g., macrophages) in the injected glands. Despite these differences in baseline immune cell compositions, no significant differences in mammary tumor development (e.g., latency, phenotype) were observed between the two models [39]. Nevertheless, this finding suggests that caution should be exercised when interpretating immune-related data, particularly in assessing how changes in immune cell populations and their functional states correlate with or influence tumor progression.

## Concluding Remarks

Intraductal injection of Ad-Cre provides a rapid and effective method for mapping normal and aberrant fates of MEC subpopulations in vivo, studying the cellular origins of breast cancer, and modeling mammary tumor initiation, progression, and metastasis. Various MEC subset-specific Ad-Cre viruses (e.g., *Ad-K8-Cre*, *Ad-K5-Cre*, Table 1) are readily available from sources such as the University of Iowa Viral Vector Core. While the intraductal injection procedure may pose technical challenges, these can be overcome by referencing published video protocols (e.g [32])., and through hands-on practice. Moreover, the intraductal injection approach is being further developed to enable Cre/loxP-based recombination locally in the mammary gland, including intraductal injection of TAT-Cre (a cell-permeant Cre recombinase) and 4-Hydroxytamixfen (a potent and active metabolite of tamoxifen that activates CreERT2) [63].

Moving forward, the adenovirus intraductal injection system can be further enhanced by incorporating the latest lineage tracing technologies. As discussed in the Introduction, reporters are a key component of lineage tracing systems. Traditional lineage tracing largely relies on fluorescent protein markers (e.g., YFP, GFP), serving as “analog” barcodes, whereas newer CRISPR-based approaches introduce “digital” barcodes, that is, CRISPR-induced random genomic mutations that can be decoded by sequencing [64–66]. Intraductal injection of adenovirus expressing Cre (e.g., under an MEC subset-specific promoter) and guide RNA (gRNA) into inducible Cas9-expressing mouse models (e.g., *Rosa26-loxP-Stopper-loxP-Cas9-EGFP* mice [67]) enables both classic “analog” lineage tracing (via GFP expression) and “digital” barcoding through Cre-mediated Cas9 expression (Fig. 1B). These barcodes can be generated from either endogenous loci (e.g., *Rosa26*) or engineered molecular recorder arrays integrated into the mouse genome. Additionally, this approach allows CRISPR-based gene editing of any target gene of interest, further expanding its utility. Alternatively, adenovirus can be engineered to express both Cas9 and gRNA directly (Fig. 1C).

By leveraging these advanced barcoding and genetic manipulation technologies, researchers can study cellular lineage relationships in the mammary gland and during mammary tumor development with greater resolution. For example, “digital” lineage tracing might help resolve the long-standing debate over unipotent versus multipotent MaSCs in the adult mammary gland [14, 24, 26]). When combined with traditional genetical engineering and adenovirus-based strategies, these methods will be instrumental in advancing stem cell, cancer biology, and developmental studies for the mammary epithelium.

## Data Availability

No datasets were generated or analysed during the current study.
